# Toxicology, biodistribution and shedding profile of a recombinant measles vaccine vector expressing HIV-1 antigens, in cynomolgus macaques

**DOI:** 10.1007/s00210-012-0793-4

**Published:** 2012-09-16

**Authors:** Clarisse Lorin, Lawrence Segal, Johann Mols, Danielle Morelle, Patricia Bourguignon, Olga Rovira, Pascal Mettens, Jérémy Silvano, Nicolas Dumey, Frédérick Le Goff, Marguerite Koutsoukos, Gerald Voss, Frédéric Tangy

**Affiliations:** 1 GlaxoSmithKline Vaccines, Rixensart, Belgium; 2 Centre International de Toxicologie, Evreux, France; 3 Texcell, Evry, France; 4 Unité de Génomique Virale et Vaccination, Institut Pasteur, CNRS URA-3015, Paris, France; 5 GlaxoSmithKline Vaccines, Rue Fleming 20, 1300 Wavre, Belgium

**Keywords:** Measles vaccine vector, HIV, Toxicology, Biodistribution, Cynomolgus macaque

## Abstract

As a new human immunodeficiency virus type 1 (HIV-1) vaccine approach, the live-attenuated measles virus (MV) Schwarz vaccine strain was genetically engineered to express the F4 antigen (MV1-F4). F4 is a fusion protein comprising HIV-1 antigens p17 and p24, reverse transcriptase and Nef. This study assessed the toxicity, biodistribution and shedding profiles of MV1-F4. Cynomolgus macaques were intramuscularly immunized one or three times with the highest dose of MV1-F4 intended for clinical use, the reference (Schwarz) measles vaccine or saline, and monitored clinically for 11 or 85 days. Toxicological parameters included local and systemic clinical signs, organ weights, haematology, clinical and gross pathology and histopathology. Both vaccines were well tolerated, with no morbidity, clinical signs or gross pathological findings observed. Mean spleen weights were increased after three doses of either vaccine, which corresponded with increased numbers and/or sizes of germinal centers. This was likely a result of the immune response to the vaccines. Either vaccine virus replicated preferentially in secondary lymphoid organs and to a lesser extent in epithelium-rich tissues (e.g., intestine, urinary bladder and trachea) and the liver. At the expected peak of viremia, viral RNA was detected in some biological fluid samples from few animals immunized with either vaccine, but none of these samples contained infectious virus. In conclusion, no shedding of infectious viral particles was identified in cynomolgus monkeys after injection of MV1-F4 or Schwarz measles vaccines. Furthermore, no toxic effect in relation to the MV vaccination was found with these vaccines in this study.

## Introduction

The development of a safe and effective prophylactic vaccine against human immunodeficiency virus type I (HIV-1) is a global health priority. During the past two decades, significant efforts have been made to develop such a vaccine. Among the few candidate HIV-1 vaccines tested in large Phase IIb or III clinical trials, only the RV144 trial, evaluating a recombinant canarypox vector prime and HIV-1 gp120 protein boost, showed a modest efficacy (31 %) against HIV-1 acquisition (reviewed by McElrath and Haynes 2010; McMichael et al. 2010). Although ultimately, a preventive vaccine against HIV-1 inducing sterile immunity would be optimal, vaccines that would reduce viral load and disease progression by induction of strong and polyfunctional T cell responses should also prove beneficial (McMichael et al. 2010).

Live attenuated measles virus (MV) vaccine strains, such as the widely used and strongly immunogenic Schwarz strain (Griffin 2007), are replicating RNA viruses (*Paramyxoviridae* family), capable of inducing long-lived antibody and memory T cell responses (Ovsyannikova et al. 2003; Vandermeulen et al. 2007). Besides being highly efficacious, these vaccines are also recognized as safe (WHO 2009), as MV replicates in the cytoplasm and does not integrate into the host cell genome. Moreover, reversion of the vaccine genome into a pathogenic form has never been observed. The experience accumulated with these vaccines in the past 50 years, and their capacity to induce both CD4^+^ and CD8^+^ T cells, render recombinant MV vectors an attractive platform for vaccines aimed to induce T cell responses specific for the HIV-1 transgene. For several of these vaccines expressing HIV-1 antigens, the immunogenicity has been preclinically demonstrated (Combredet et al. 2003; Guerbois et al. 2009; Liniger et al. 2009; Lorin et al. 2004).

We constructed the HIV-1 vaccine candidate MV1-F4, using an in vivo replication-competent MV vector (Combredet et al. 2003) derived from the Schwarz vaccine strain, to generate recombinant MV expressing the F4 antigen. F4 is a fusion protein comprising the clade B viral antigens p17, p24, reverse transcriptase and the regulatory protein Nef. Combined with AS01 (a liposome-based Adjuvant System containing 3-*O*-desacyl-4′-monophosphoryl lipid A (MPL) and QS21; Garçon et al. 2007), this F4 antigen was shown to induce potent polyfunctional CD4^+^ T cell responses in HIV-seronegative volunteers (Van Braeckel et al. 2011).

We conducted a study of the biodistribution, shedding and single- and repeated-dose toxicity of one or three intramuscular (IM) immunizations with MV1-F4 in cynomolgus macaques. The potential intrinsic toxicity of MV1-F4 was studied, as well as the potential immune-mediated toxicity resulting from the host response to the vaccine. The resulting toxicity and biodistribution profiles, including any target organs identified, could be used to guide clinical safety monitoring, while the shedding profile is crucial for determining the potential of infectious viral dissemination by future vaccine recipients, and thus of person-to-person transmission of the virus. The biodistribution, shedding and toxicity profiles of the MV1-F4 vaccine were compared to those of either the reference vaccine (the live attenuated monovalent Schwarz MV vaccine Rouvax), or saline.

Humans are the natural hosts of MV. Measles pathogenesis has traditionally been studied in non-human primates as no suitable alternative models exist. Live attenuated MV vaccines are generally non-infectious in rodents, except for cotton rats (in which MV replication is restricted to the lungs) and transgenic mice (which reproduce only limited aspects of MV pathogenesis) (de Swart 2008). In the current study, we used (MV-seronegative) cynomolgus monkeys. This species is highly sensitive to MV infection and able to develop pathologic lesions and clinical symptoms comparable to those in human MV infections (Kobune et al. 1996). Moreover, their size permits the administration of a full human vaccine dose.

As none of the known Schwarz strain virus traits, including viral envelope proteins, were altered for the construction of the MV1-F4 vector, we hypothesized that the toxicological profile, tropism and shedding capacity of MV1-F4 is similar to those of the parental strain (as represented by the reference vaccine). In addition, we hypothesized that both vaccine viruses have the potential to spread into a large variety of organs, as attenuated MV strains can use the CD46 receptor (which is ubiquitously expressed in both humans and cynomolgus macaques; Sakurai et al. 2008), in addition to the receptors used by wild-type MV (Dorig et al. 1993). Wild-type MV strains use primarily the signaling lymphocyte activation molecule (SLAM/CD150) expressed on certain immune cells, and likely other low-affinity receptors to enter CD150-negative epithelial cells (Tatsuo et al. 2000b; Watanabe et al. 2010). As a result, these strains replicate predominantly in lymphoid organs and epithelial tissues (Griffin 2007; Takeda 2008). Consequently, MV1-F4 and Rouvax were expected to spread not only to the latter organs and tissues, but also to other parts of the body.

## Materials and methods

### Vaccines

The MV vector pTM-MV-Schw that was used to construct the MV1-F4 recombinant virus has been described previously (Combredet et al. 2003). This vector contains an infectious MV cDNA corresponding to the anti-genome of the Schwarz MV vaccine strain. An additional transcription unit (ATU) was inserted into pTM-MV-Schw in order to sub-clone the F4 fusion protein sequence. F4 has been described previously (Van Braeckel et al. 2011) and comprises HIV-1 subtype B antigens p24 (BH10), RT (HXB2), Nef (Bru-Lai) and p17 (BH10). The ATU contains a cloning-site cassette inserted into a copy of the original N-P intergenic region of the MV genome. This region contains the *cis*-acting sequences required for transcription of the viral P gene, enabling the recombinant MV to express the F4 protein like a measles gene. The ATU was introduced into the plasmid backbone by site-directed mutagenesis between the MV P and M genes, resulting in the plasmid pTM-MVSchw-ATU2_F4co_mut. MV1-F4 virus was rescued from the pTM-MVSchw-ATU2_F4co_mut plasmid using a helper cell-based system developed at the Institut Pasteur. Briefly, helper HEK293 cells expressing both the T7-RNA polymerase and the Schwarz MV N and P proteins were co-transfected with the pTM-MVSchw-ATU2_F4co_mut cDNA and a plasmid expressing the Schwarz MV polymerase L. Subsequently, transfected HEK293-T7-MV helper cells were gently harvested and co-cultured with MRC-5 cells for the amplification of the MV1-F4 virus. Virus titers were determined by endpoint titration on Vero cells and were expressed as 50 % cell culture infectious dose (CCID_50_)/ml.

The selected route of administration (IM) and dose were identical to those intended to be used in the first MV1-F4 clinical trial, with the dose-level representing the highest intended human dose. One-dose and three-dose schedules were used, with the three-dose schedule representing the number of doses to be used in the clinical study, plus one. One milliliter of MV1-F4 vaccine (viral titer 1.6 × 10^4^ CCID_50_/ml) was injected intramuscularly. The reference vaccine, the commercial (Schwarz) MV vaccine Rouvax (Sanofi-Aventis, Paris, France) was used according to the manufacturer’s procedure for IM administration, with each vaccine dose containing 3.9 × 10^3^ CCID_50_ of the Schwarz measles vaccine. This virus titer was determined with the same assay as was used for the MV1-F4 vaccine (endpoint titration on Vero cells). All injections were administered in the thigh muscle.

### Animals and husbandry

The study included purpose-bred cynomolgus monkeys (*Macaca fascicularis*), aged either 2–3 years (males and females) or 7–12 years (sexually mature males), obtained from Noveprim Ltd (Port Louis, Mauritius) that were seronegative for anti-MV antibodies. During the full experimentation period, the animals were housed in a dedicated primate unit under controlled environmental conditions. Due to the biosafety level of the MV1-F4 candidate vaccine (class 2, group II), they were housed individually in stainless steel cages (level A2, L2 confinement) and appropriate precautions were established. The study was conducted in compliance with the European regulations regarding the protection of animals used for experimental and other scientific purposes, and an ethical committee reviewed the study plan before the initiation of the study.

### Study design

The study design followed the guidelines published by the European Medicines Agency (EMA) as well as other relevant guidelines (EMEA 1995, 2000; WHO 2005). All experiments were performed under good laboratory practices (GLP) conditions.

Animals were inoculated and monitored at the laboratories of the Centre International de Toxicologie (CIT; Evreux, France). Of the 42 monkeys included in the study, 18 young males and 18 young females were allocated to treatment groups 1–6 (Table 1). Animals were allocated to groups (by sex) using a computerized randomization procedure (CITOX software, developed in-house at CIT, Evreux, France). In addition, six sexually mature males (three per group) were allocated to treatment groups 7 and 8 using the same randomization procedure, and were only used for collection of semen and peripheral blood mononuclear cells (PBMC), as well as for selected clinical observations (body weight, body temperature and food consumption). Treatments included 3.9 × 10^3^ CCID_50_ of Rouvax, 1.6 × 10^4^ CCID_50_ of MV1-F4 vaccine, or saline, and were administered according to either a one-dose schedule (injection at day 1) or a three-dose schedule (injections at days 1, 29 and 57). Monkeys were monitored clinically until sacrifice (groups 1–6) or until return to laboratory stock at day 85 (groups 7 and 8). Upon completion of the observation period, animals of groups 1–6 were sedated with ketamine hydrochloride (Imalgène, Mérial, Lyon, France), then anesthetized with thiopental and sacrificed by exsanguination. Necropsy was performed at two time-points, either within the expected peak of viremia (10 days after the first vaccine dose (day 11) for groups 1–3) or when clearance of infectious MV was expected to have occurred (represented by 28 days after the third dose [day 85] for groups 4–6) (Auwaerter et al. 1999; Pan et al. 2005; Permar et al. 2003). Single- and repeated-dose toxicity was assessed at various time points (Table 2). Shedding and post-mortem analyses were conducted at Texcell (Evry, France). All other procedures were conducted at CIT.

**Table 1:** Study design

Group	No. of animals	Immunization	Dose level (CCID_50_)	Time points Immunization	Time points for necropsy and biodistribution analysis*	Time point of return to stock	Shedding analysis^a^	
							Time point	Collected samples^b^
1	3 M, 3 F	NaCl	0	Day 1	Day 11		Days 0, 4, 11	(A)
2	3 M, 3 F	MV1-F4	1.6 × 10^4^	Day 1	Day 11		Days 0, 4, 11	(A)
3	3 M, 3 F	Rouvax	3.9 × 10^3^	Day 1	Day 11		Days 0, 4, 11	(A)
4	3 M, 3 F	NaCl	0	Days 1, 29, 57	Day 85		Days 0, 4, 11, 29, 56, 60, 67, 85	(A)
5	3 M, 3 F	MV1-F4	1.6 × 10^4^	Days 1, 29, 57	Day 85		Days 0, 4, 11, 29, 56, 60, 67, 85	(A)
6	3 M, 3 F	Rouvax	3.9 × 10^3^	Days 1, 29, 57	Day 85		Days 0, 4, 11, 29, 56, 60, 67, 85	(A)
7	3 mature M	MV1-F4	1.6 × 10^4^	Days 1, 29, 57		Day 85	Days 0, 4, 11, 29, 56, 60, 67, 85	(B)
8	3 mature M	Rouvax	3.9 × 10^3^	Days 1, 29, 57		Day 85	Days 0, 4, 11, 29, 56, 60, 67, 85	(B)

*M/F* male/female, *CCID*_*50*_ 50 % cell culture infectious dose

^a^Pretreatment = day 0; days 4, 11 and 29 = 3, 10 and 28 days after the first dose, respectively; day 56 = 1 day prior to the third dose; days 60, 67 and 85 = 3, 10 and 28 days after the third dose, respectively

^b^(A) PBMC, serum, throat swabs, saliva, nasal swabs, urine and vaginal secretion; (B) PBMC, semen

**Table 2:** Toxicology recording time points

		Clinical examinations^a^				Clinical pathology		Gross pathology, organ weights, histopathology^e^
Study day	Treatment	Dermal reactions^b^	Body temperature	Electrocardiography^c,d^	Ophthalmology^c^	Blood collection^e,f^	Urine collection^c^	
Pre			×	×	×	×	×	
1	×	×		×				
2		×					×	
4						×		
8			×					
9			×					
10			×					
11^*g*^			×			×		×
29	×	×				×		
30		×						
56						×		
57	×	×		×	×			
58		×					×	
60						×		
67			×			×		
85^*g*^					×	×		×

^a^Body weights: recorded at pre-treatment, days 1 and 4 and then once weekly until study end. Food consumption: estimated daily from 5 days before start throughout the study

^b^Dermal observations at the injection sites were recorded at 3 and 24 h after each vaccination

^c^Performed for groups 4–6 only

^d^ On days 1 and 57, tests were done just before and 1 h post immunization. A test at day 85 was done before sacrifice for only one animal

^e^Blood sampling for clinical pathology performed for groups 1–6 only

^f^At day 55 or day 56, blood samples for haematology, coagulation parameters and blood biochemistry were taken before immunisation. On day 85, samples were taken before necropsy

^g^Day 11, day 85 *=* time points of termination of the animals of groups 1–3, groups 4–6 respectively

### In-life procedures

Animals were monitored at least twice daily for mortality, morbidity and clinical signs of toxicity. Toxicological parameters included dermal reactions at the injection sites, body weight, food consumption, rectal body temperature, electrocardiography and ophthalmology. Complete clinical examinations were performed pre-treatment and thereafter once weekly. Sedation, used for electrocardiography, ophthalmology and occasionally for weighing, was done by IM administered ketamine hydrochloride (Imalgène).

At 3 and 24 h after each immunization, dermal reactions, including edema and erythema formation, were evaluated using the Draize scale, and any other lesions were noted. Reactions persisting for 48 h after immunization were evaluated daily until disappearance.

Electrocardiographic examinations were performed using a Cardioline Delta 3 Plus and Cardiovit AT-6 (Schiller AG, Baar, Switzerland) with standard leads I–III, starting with determining the heart rate, PQ and QT intervals and the QRS-complex duration on lead II.

Ophthalmology included assessment of pupillary light reflexes using tropicamide (Mydriaticum, Théa, Clermont-Ferrand, France), examination of appendages, optic media and fundus by indirect ophthalmoscopy (Oméga 180, Heine, Germany) and of anterior segments and lenses (portable slit-lamp biomicroscope, model SL-15; Kowa, Japan).

### Clinical pathology

Peripheral blood samples were collected without sedation at different time points after the first and the third dose (Table 2), in tubes containing EDTA, sodium citrate or lithium heparin (for haematology, coagulation parameters or blood biochemistry, respectively).

Haematology (i.e., erythrocyte count, haemoglobin (HB), mean and packed cell volumes, mean cell HB concentration, mean cell HB, thrombocytes, leucocytes (differential) and reticulocytes) was determined by ADVIA 120 haematology analyser (Siemens, Saint-Denis, France). Leukocyte differential analysis (with cell morphology) was assessed in blood smears stained with May–Grünwald–Giemsa. Coagulation parameters (i.e., prothrombin time, activated partial thromboplastin time and fibrinogen) were measured with an ACL300 coagulation analyzer (Beckman Coulter, Instrumentation Laboratory, France). Complete blood biochemistry was assessed by the ADVIA 1650 Chemistry System (Siemens) using whole blood.

Urinalysis (including volume, pH, specific gravity, proteins, glucose, ketones, bilirubin, nitrites, blood (HB) and urobilinogen) was done using a Clinitrek 500 urine chemistry analyzer (Siemens).

### Anti-MV antibody response

Blood samples for assessment of humoral responses were taken at prevaccination and days 11, 29, 56, 67 and 85. Anti-MV humoral responses in sera were measured using an anti-MV enzyme-linked immunosorbent assay (ELISA) adapted from the commercial immunoassay Enzygnost Anti-Measles Virus/Ig (Dade-Behring, Illinois, USA). A peroxidase-labelled anti-monkey immunoglobulin (Ig) secondary antibody (Rockland, Pennsylvania, USA) was used in the assay. Animals were considered to be responders if they were seropositive for anti-MV antibodies, i.e., if the absorbance (measured at 490/620 nm) exceeded the cut-off value, for a serum dilution of 1/500. The cut-off values (percentile 100) were determined per gender for sera collected prior to vaccination (serum dilution 1/500), and were found to be equal to 1.74 and 1.22 for sera from female and male monkeys, respectively.

### Gross pathology, organ weights and histopathology

At necropsy, organs were weighed and subjected to macroscopic and microscopic examination. The selection of organs to be examined for gross pathology and histopathology analyses followed the applicable European and international guidelines (EMEA 1995, 2000; WHO 2005). Of this selection, the following organs were weighed: adrenals, brain, epididymides, heart, kidneys, cervical and iliac lymph nodes, liver, lungs with bronchi, ovaries, pituitary gland, prostate, spleen, testes, thymus, thyroid with parathyroid and uterus. Ratios of organ weight to body weight (at necropsy) were calculated. For gross pathology, the external surface of the body, orifices, the neck with organs and tissues, the cranial cavity with surfaces of the brain and spinal cord and the thoracic, abdominal and pelvic cavities with their contents were examined. For histopathological examination, 4-μm**-**thick sections of tissues were stained with haematoxylin and eosin.

### Sample processing for biodistribution and shedding analyses

The selection of organs to be examined for biodistribution analyses followed the applicable European and international guidelines (EMEA 1995, 2000; WHO 2005). For biodistribution analyses, tissue samples (3 × 3 × 3 mm^3^) were dissected out, rinsed with cold phosphate-buffered saline (PBS) and snap-frozen in cryotubes in liquid nitrogen.

For shedding analyses, selected excretions and biological fluids (i.e., semen [only for groups 7 and 8], serum, urine, throat and nasal swabs, saliva, vaginal secretion and faeces) and PBMC were collected. Throat swabs, saliva, nasal secretions and vaginal secretions were conserved in M4RT tubes (Remel, Oxoid, Cedex, France) at −70 °C. Semen and faeces were directly frozen in cryotubes and conserved at −70 °C. Immediately after collection, urine was centrifuged (800 × *g*, 10 min, 4 °C) and the pellet was resuspended in PBS and stored at −70 °C. PBMC were isolated from EDTA blood samples by gradient centrifugation and frozen in PBS (−70 °C). To obtain serum, blood was collected in plain tubes, incubated (20 min, room temperature) and centrifuged (3,000 × *g*, 10 min, 4 °C).

### RT-qPCR analysis

Samples of organs, tissues, excretions and biological fluids were used for RNA extraction and MV N-specific, quantitative reverse transcriptase (RT) PCR analysis (RT-qPCR). Briefly, cDNA was synthesized from the RNA templates, and then amplified by qPCR specific for the MV N gene. This gene is identical in both vaccine viruses used in this study.

Frozen organ samples were homogenized using a Tissue Lyser (30 Hz for ≥2 min; Qiagen, Valencia, CA) and kept on ice. Samples were then lysed using 1 ml of Qiazol lysis reagent (Qiagen). Frozen PBMC, excretions and biological fluids were directly (for urine, serum, throat swabs, nasal swabs and vaginal secretion), or with a 1/45 dilution factor (for semen) submitted to RNA extraction. Faeces was resuspended into PBS to 1/10 (v/v), centrifuged, and the supernatant was filtered (0.22 μm).

Total RNA was extracted from the samples using an RNeasy 96 kit with the Biorobot 3000 (both Qiagen) and reagents and protocols recommended by the manufacturer. Briefly, the lysed samples in 0.2 ml of chloroform were homogenized (15 s), left at room temperature for 2–3 min and centrifuged (12,000 × *g*, 15 min, 5 °C). The aqueous phase was transferred to 96-well plates and placed on the Biorobot for automatic extraction (by vacuum or centrifugation). Ethanol (600 μl, 70 % (v/v)) was added to each well. After mixing, samples were placed in the column for RNA adsorption on the membranes. After three wash steps (once with 800 μl RW1 buffer and twice with 800 μl RPE buffer), columns were centrifuged (5,600 × *g*, 10 min). RNA was then eluted with 60 μl elution buffer (RNase free water) and stored at temperatures below −70 °C.

RT and qPCR were performed using the AgPath-ID One-step RT-PCR kit (Applied Biosystems, Inc., Foster City, CA) and the LightCycler 480 and software version 1.5.0.39 (Roche Applied Sciences). The forward primer was MeN_TMF(5′-GCGAGAGCTGCCCATCTTC), the reverse primer was MeN_TMR (5′-ACTCCGTTGCAGTGTCAATGTC) and the forward MeN_TMP probe was (6-FAM) ~ AACCGGCACACCC ~ (NFQ), with the fluorogenic probe located in the region bracketed by forward and reverse primers. Cycling conditions were 45 °C for 10 min, 95 °C for 10 min, and 45 cycles of 95 °C for 15 s and 60 °C for 45 s, followed by 40 °C for 2 min. The standard curve and RT-qPCR parameter determination was performed using an in vitro transcript RNA template with the sequence of the N gene of the measles vector. MV1-F4 suspension (GSK; EF4MA001A, viral titer 4.2 log CCID_50_/ml, 7.2 log genome equivalent (geq)/ml, diluted to 10^4^ copies geq), was used as positive extraction control. Negative (RNase/DNase free H_2_O) and positive extraction controls were run in quadruplicate and duplicate reactions, respectively. MV1-F4 RNA recovery was assessed by N-specific RT-qPCR after spiking each type of tissue with MV1-F4 at various concentrations, and ranged from 90 % to 100 %. The presence of MV1-F4 RNA was considered positive if the measurements exceeded the limit of detection (LOD) of 100 geq/reaction, and if the acceptance criteria (error <0.2, efficiency > 90 % and crossing point <45) were fulfilled. The LOD was defined as the lowest amount of copies that can be detected (but not necessarily quantified as an exact value) with 95 % probability. All samples were tested once.

### In vitro infectivity and immunofluorescence assays

All samples in which a RT-qPCR signal was detected (either confirmed [above LOD], or at a very low level [below LOD]) were submitted to an in vitro infectivity assay. The presence of infectious viral particles for all fluids except semen was considered positive for values exceeding the LOD of 10 CCID_50_/well (with 95 % probability). Nevertheless, the assay was designed to detect one infected cell that propagates the virus to its adjacent cells, resulting in a plaque forming unit (as aided by the presence of agarose in the culture medium). Therefore, it was assumed that the system was able to detect 1 CCID_50_/well (but with a probability of detection of less than 95 %). However, for this to occur at least 10 CCID_50_ should be present in the inoculum, in order to allow the adsorbtion onto the cells. For semen, validation of the infectivity assay could not be conducted due to high bacterial contamination.

Vero cells were cultured in Dulbecco’s modified Eagle’s medium (DMEM)–5 % fetal calf serum (FCS)–4 mM glutamine. Before seeding the cells, a sterilized (ethanol, UV) coverslip (18 × 18 mm^2^) was placed in each well. One day before inoculation, 6-well plates were seeded with 4 × 10^5^ Vero cells/well. Biological fluid samples were diluted (1: 15.625 for serum, 1:10 for all other fluids) in dilution medium (DMEM–1 % antibiotics) and 700 μl of each fluid sample was added to Vero cells for 2 h of inoculation (37 °C, 5 % CO_2_). Inoculum was then removed and 2 ml of survival medium (DMEM–2 % FCS–4 mM of glutamine–1 % antibiotics) supplemented with 1 % (v/v) low-melting agarose was added. The plates were incubated (37 °C, 5 % CO_2_) for 6 days. As a positive control, Vero cells were inoculated with100 CCID_50_ of MV1-F4 viral suspension (as described above) and diluted in 700 μl dilution medium. As a negative control, Vero cells were inoculated with 700 μl dilution medium.

The presence of infectious viral particles was revealed by immunofluorescence assay (IFA), using an antibody directed against the measles N protein. The acceptance criteria of the assay were the absence or presence of fluorescence, respectively, in the negative or the positive controls.

After 6 days of incubation, the culture medium was removed and 2 ml of 4 % paraformaldehyde was added (15 min, 5 °C). The slides were washed with PBS and incubated overnight with 2 % goat serum at 5 °C. Next, 100 μl of the primary antibody (mouse anti-MV-N monoclonal antibody [# MAB8906; Chemicon, Temecula, CA, USA]), diluted 1:200 in PBS–2 % goat serum–0.1 % saponin, was added to each well (60 min, room temperature). After washing twice with PBS, 100 μl of the secondary antibody (goat anti-mouse F(ab′)_2_-Cy3 [# LU1513615; Jackson ImmunoResearch, West Grove, PA, USA]) diluted to 1:500 in PBS–2 % goat serum–0.1 % saponin, was added to each well. After incubation (45 min, room temperature), coverslips were washed twice with PBS, rinsed with water, mounted on a slide using Vectashield DAPI mounting medium (Vector Laboratories, Peterborough, UK) and inspected under fluorescence microscope. Positivity or negativity was checked by the presence or absence of fluorescence, respectively.

### Statistical analysis

The following sequence was used for statistical analyses of body weights, body temperatures and electrocardiography, haematology, blood biochemistry and urinalysis data. A test of normality using the Kolmogorov–Lilliefors test was performed, followed by log-transformation of the data if normality assumptions were not satisfied. Assessment of homogeneity of variance was performed using the Bartlett test. If the homogeneity of variance was not rejected, the treatment groups were compared to the control group using the Dunnett test, in other cases the Dunn test was applied. The same procedure, but without log-transformation of the data, was performed for the statistical analysis of organ weights using PathData software (version 6.2b5). Statistical significance was expressed as *p* < 0.05 or *p* < 0.01.

## Results

### Clinical monitoring

No mortality or morbidity was observed in any of the groups (Table 3). The only clinical effect recorded for a vaccine group was marked dehydration (without diarrhoea) in a Rouvax-treated male on days 56–62, which resolved within the following 2 days. Scab formation was noted in one Rouvax-treated male each of the one-dose and three-dose groups. These effects were incidental, self-limiting and of a mild-to-moderate nature, and therefore considered toxicologically irrelevant. In the controls of group 4, incidental vomiting (day 1; one male) and very slight erythema (days 1–3, one female) were noted.

**Table 3:** Toxicology results

Parameters	MV1-F4	Rouvax	Controls
Mortality	None observed	None observed	None observed
Clinical observations			
Local reactogenicity			
-Dermal observations IS	None	Scabs (2 M, groups 3 and 6)	Slight erythema (1 F, group 4, days 1–3)
Systemic reactogenicity			
-Body weights	No changes	No changes	No changes
-Food consumption	No vaccine-related effect	No vaccine-related effect	No changes
-Body temperature	No vaccine-related effect	No vaccine-related effect	No changes
-Electrocardiography	No vaccine-related effect	No vaccine-related effect	Sinusal arrhythmia (1 F) pretreatment
-Ophthalmology	No vaccine-related effect	No vaccine-related effect	Hypopigmented fundus (2 F)
-Other effects	None observed	Dehydration (1 M, days 56–64)	Vomiting (1 M, group 4, day 1)
Clinical pathology			
-Haematology	No vaccine-related effect	No vaccine-related effect	No changes
-Coagulation parameters	Mean aPTT time in males shorter than in controls (day 56)	No vaccine-related effect	No changes
-Blood biochemistry	No vaccine-related effect	Mean CK value in males higher than in controls (day 85)	No changes
-Urinalysis	No vaccine-related effects	No vaccine-related effects	No changes
Gross pathology	No vaccine-related macroscopic findings	No vaccine-related macroscopic findings	No changes
Organ weights	- Elevated absolute and relative spleen weights on days 11 (in males) and day 85 (in both genders, statistically significant in Rouvax-treated females).		No changes
	-Elevated mean absolute and relative weights of cervical and iliac lymph nodes, mainly in treated males (both vaccines; days 11 and 85).		
	-Lowered absolute and relative thymus weights in treated males (both vaccines; days 11 and 85).		
Histopathology	-Increased size of white pulp for both sexes on day 11 (MV1-F4) and day 85 (both treatments, corresponding with higher spleen weights).		Increased size of white pulp in females (day 11).
	-Increased sizes/numbers of germinal centers in mandibular lymph nodes in MV1-F4 treated males (day 11).		Increased sizes/numbers of germinal centers in mandibular lymph nodes in females (day 85).

Changes reported as compared to baseline

*M/F* male/female, *IS* Injection site, *aPTT* activated partial thromboplastin time, *CK* creatine kinase

For both sexes, mean body weights of vaccine and control groups were not significantly different and remained virtually constant, with minor increases between days 0 and 85. Apart from scheduled fasting days prior to procedures, the individual food consumption was generally 75–100 % of the daily ration across groups. On days when animals were sedated, the consumption was typically 25–50 % of the daily ration, and occasionally 0 % (on days 1 and 57 for nearly all females of groups 4–6, and on day 84 for two Rouvax-treated males). These fasting periods had no impact on body weights and were similar across control and vaccine groups, and thus considered to be unrelated to immunization.

Mean rectal temperatures of groups of vaccinated males were not significantly different from those of the control groups at all time points. For females, the sporadically noted statistical differences between groups remained within the ranges commonly recorded in animals of this age housed in similar experimental conditions. Consequently, no treatment-related effects on body temperature were observed in the study.

There were no treatment-related abnormalities in the qualitative and quantitative electrocardiography parameters. Although low-amplitude R and P waves and high- or low-amplitude, biphasic and negative T waves were noted, these findings were isolated, observed both prior to and post treatment, and occurred in both controls and immunized groups. They were also commonly observed in non-treated cynomolgus monkeys kept under similar laboratory conditions. No statistical differences in quantitative parameters (PQ, QRS and QT intervals and heart rates) were recorded between any of the groups. Sinusal arrhythmia was observed prior to treatment in one female in the control group.

No relevant ophthalmological findings were observed in any treatment group. In two control females, a hypopigmented fundus was noted at all three observation time points.

### Clinical pathology

No treatment-related effects were noted for haematological, blood clinical chemistry and urinalysis parameters in any of the vaccine groups. At all time points, values for control and immunized animals of both genders were similar. On day 56, the mean activated partial thromboplastin time (aPTT) for MV1-F4-treated males was slightly (15 %) shorter than for control males (i.e., 17.4 vs. 20.4 s; *p* < 0.01), which was not observed for females of this group or for other treatment groups. Prothrombin times (PT) were unaffected by immunization. For blood biochemistry parameters, the only relevant finding was a higher mean creatine kinase (CK) activity in Rouvax-treated males compared to control males (i.e., 3,760 vs. 1,010 IU/l; *p* < 0.05) on day 85, due to elevated values recorded for two animals.

### Anti-MV humoral response

**Fig. 1:**
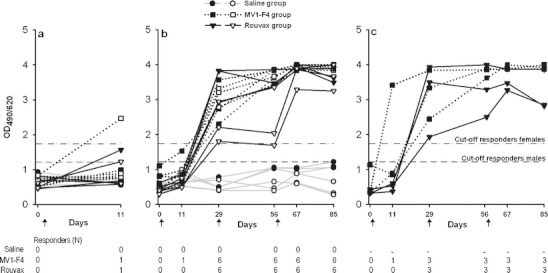
Anti-MV antibody levels and seroconversion rates. Serum levels of anti-MV antibodies were measured prior to or after injection of MV1-F4, Rouvax or saline. Antibody levels were expressed as the ratio between the optical density (*OD*) at 490 and at 620 nm. Animals were considered to be responders if the absorbance exceeded the cut-off value, for a serum dilution of 1:500. Each line represents one monkey. Plain and open symbols represent male and female monkeys, respectively. **a** Young animals (*N* = 6/group) were immunized at day 1 and sacrificed at day 11 (groups 1, 2 and 3). Blood samples were taken at days 0 and 11. **b** Young animals (*N* = 6/group) were immunized at days 1, 29 and 57, and sacrificed at day 85 (groups 4, 5 and 6). Blood samples were taken at days 0, 11, 29, 56, 67 and 85. **c** Sexually mature animals (*N* = 3/group) were immunized at days 1, 29 and 57 (groups 7 and 8). Blood samples were taken at days 0, 11, 29, 56, 67 and 85

To evaluate the humoral responses induced by both vaccines, anti-MV (Ig) antibody responses were measured in sera. Ten days after a single dose (day 11), responses were undetectable in the majority (87 %) of immunized animals (Fig. 1). However, a 100 % seroconversion rate was observed in the animals of the three-dose Rouvax or MV1-F4 groups (groups 5–8) from 28 days after the first dose (day 29) onwards. This suggests that the anti-MV humoral responses may not have been fully developed at day 11, as confirmed by the kinetics of these responses in the vaccinated animals.

### Viral shedding in body fluids

To assess the potential release of infectious MV1-F4 viral particles in PBMC, excretions and biological fluids (including serum, urine, throat- and nasal swabs, saliva, vaginal secretions, semen and faeces), samples were first tested for the presence of MV viral sequences by N-specific RT-qPCR assay. All samples in which viral RNA was detected, even if below the defined LOD, were then tested further using an in vitro infectivity assay.

Of the eight time points at which shedding analysis was performed, MV viral RNA was only detected at day 11 in some of the vaccine groups. At this time point, all signals in animals of all vaccine groups were below the LOD (Table 4), with the exception of one positive result in the three-dose Rouvax group. In the MV1-F4 groups, viral RNA was detected (below the LOD) in faeces from two males of the one-dose group and in urine and vaginal secretions from one female of the three-dose group. In the Rouvax groups, viral RNA was detected in throat secretions (above the LOD, three-dose group), in faeces, throat swabs, vaginal secretion and serum (below the LOD, three-dose group), in nasal secretions (below LOD, both groups) and in PBMC (below the LOD, three-dose group and group of mature males). No positive results were detected in any of the samples of the control groups or in saliva samples of any treatment group.

**Table 4:** Detection of MV RNA in biological fluids/excretions by RT-qPCR at day 11

Treatment group		Detection of viral RNA				Result infectivity assay
		Signal < LOD^a^		Positive signal (+)^b^		
		Animals (N)	Body fluid	Animals (N)	Body fluid	
NaCl	Group 1	–	–	–	–	–
	Group 4	–	–	–	–	–
MV1-F4	Group 2	2 M	Faeces	–	–	Negative
	Group 5	1 F	Urine, vaginal secretion	–	–	Negative
	Group 7	–	–	–	–	–
Rouvax	Group 3	1 F	Nasal swab	–	–	Negative
	Group 6	1 M	Serum, faeces, PBMCs	1 M	Throat swab	Negative
		1 M	Nasal swab, faeces			
		1 F	Throat swab, vaginal secretion			
	Group 8	1 MM	PBMCs	–	–	Negative

*(M)M/F* (mature) male/female; *N* no. of animals with positive signal in sample/no. of tested animals in that group

^a^<LOD: RNA signal detected but the number of copies was less than the defined LOD of 100 genome equivalent (geq)/reaction (with 95 % chance to detect a positive signal).

^b^+: positive signal, i.e., signal > defined LOD of the RT-qPCR assay (with 95 % probability)

In the in vitro infectivity assay and at the LOD of 10 CCID_50_/well, no infectious viral particles were recovered from any sample in which viral RNA had been detected, with acceptance criteria (i.e., negative and positive controls showing respectively negative and positive results) being fulfilled.

### Biodistribution in organs and tissues

The potential presence of viral RNA in organs and tissues from vaccinated monkeys was detected by RT-qPCR specific for the N gene. This gene is identical in both vaccine viruses used in this study.

At day 11, MV viral RNA was detected in a large number of animals and tissues from both vaccine groups (Table 5), confirming the expected time point of the peak of viral replication. In animals of both one-dose vaccine groups, viral RNA was mainly detected in secondary lymphoid organs (lymph nodes, spleen, Peyer’s patches) and in the non-lymphoid tissues of the intestine (with the majority of signals detected above the LOD) and to a lesser extent in the liver, trachea, larynx and urinary bladder. Viral RNA was also detected in few animals in lungs, eyes, adrenals (for animals in both vaccine groups), in ureters and salivary glands (for the MV1-F4 group) and in several other organs for the Rouvax group. Importantly, no viral RNA was detected in the brain, cerebellum, cerebrum, spinal cord regions, thalamus and hypothalamus of any animal. No gender-determined patterns were detected.

**Table 5:** Detection of MV RNA in organs and tissues by RT-qPCR

Organs/tissues	Day 11		Day 85	
	MV1-F4 — 1 dose (Group 2) *N*	Rouvax — 1 dose (Group 3) *N*	MV1-F4 — 3 doses (Group 5) *N*	Rouvax — 3 doses (Group 6) *N*
Adrenals	1/6 < LOD	1/6 < LOD	–	–
Large intestine (caecum/colon/rectum)	2/6+; 2/6 < LOD	3/6+; 1/6 < LOD	–	1/6+
Small intestine (ileum/jejunum/duodenum)	2/6+; 1/6 < LOD	4/6+; 1/6 < LOD	–	1/6 < LOD
Eye: retina + lid	1/6 < LOD	1/6 < LOD	–	–
Gall bladder	–	2/6+	–	–
Kidneys	–	1/6+	–	–
Larynx	1/6+	2/6+; 1/6 < LOD	–	1/6 < LOD
Liver	3/6 < LOD	1/6+; 5/6 < LOD	–	–
Lungs with bronchi	1/6+; 1/6 < LOD	1/6 < LOD	–	–
Lymph nodes (cervical)	4/6+	5/6+	1/6+	2/6+; 1/6 < LOD
Lymph nodes (iliac)	4/6+; 1/6 < LOD	4/6+	3/6+; 1/6 < LOD	2/6+; 2/6 < LOD
Lymph nodes (mandibular)	4/6+	5/6+	2/6 < LOD	4/6+
Lymph nodes (mesenteric)	3/6+; 1/6 < LOD	5/6+	1/6 < LOD	3/6+; 2/6 < LOD
Lymph nodes (popliteal)	2/6 < LOD	2/6+; 1/6 < LOD	1/6+	3/6+
Mammary gland area	–	1/6 < LOD	–	–
Mesenteric artery	–	1/6+; 1/6 < LOD	–	–
Pancreas	–	2/6 < LOD	–	–
Peyer’s patches	2/6+; 1/6 < LOD	4/6+	–	1/6+; 1/6 < LOD
Salivary glands	2/6 < LOD	–	–	–
Skin	–	1/6 < LOD	–	–
Pia maters (cervical/lumbar/thoracic)	–	1/6 < LOD	–	–
Spleen	4/6+; 1/6 < LOD	5/6+	1/6+; 2/6 < LOD	3/6+; 1/6 < LOD
Sternum with bone marrow	–	2/6 < LOD	–	–
Thymus	–	1/6+	–	–
Tongue	–	1/6 < LOD	–	–
Trachea	2/6 < LOD	2/6+; 1/6 < LOD	–	1/6 < LOD
Ureters	1/6+	–	–	–
Urinary bladder	1/6+; 2/6 < LOD	1/6 < LOD	–	–
Uterus corpus + cervix	–	1/3 < LOD	–	–
Vagina	–	1/3 < LOD	–	–

No detections were made in the remaining organs (aorta, brain, cerebellum, cerebrum, epididymides, femoral bone with articulation, heart, hypothalamus, injection sites, knee joint, optic nerve, oesophagus, ovaries, oviducts, pituitary gland, prostate, sciatic nerve, seminal vesicles, skeletal muscle, spinal cord regions, stomach (fundus), testes, thalamus, thyroids and parathyroids) for these groups and time points

*N* no. of animals with positive signal in tissue sample/no. of tested animals in that group; *+/−*: + = positive signal (signal > defined limit of detection [LOD] of the RT-qPCR assay [with 95 % probability]). – : no RNA signal detected; <LOD: RNA signal detected but the number of copies was less than the defined LOD of 100 geq/reaction (with 95 % chance to detect a positive signal)

At day 85 (28 days after the third dose), viral RNA was still detectable in the spleen and lymph nodes of some animals (both vaccine groups), and also in Peyer’s patches and the intestine (Rouvax group). Low signals were also detected in the larynx and the trachea (Rouvax group).

No viral RNA was detected in the controls at both time points of necropsy.

### Gross pathology, organ weights and histopathology

Necropsy after either one or three injections revealed no macroscopic findings attributable to either treatment. Recorded findings were sporadic or common for cynomolgus monkeys and therefore considered toxicologically irrelevant.

At day 11, the mean absolute and relative spleen weights of males of both vaccine groups tended to be higher than for control males (without reaching statistical significance), which was only observed to a minor extent for the females of these groups (Table 6). Similar effects were observed for the cervical lymph nodes. For the iliac lymph nodes, which were the draining lymph nodes for the injection sites (the thighs), there was a slight tendency for higher weights in all Rouvax-treated animals. Mean thymus weights tended to be lower in both treated male groups than in control groups, possibly due to high absolute and relative weights of one control male (129 % and 118 % of group mean values, respectively). No clear effects of immunization were observed on the thymus weights of females of any group.

**Table 6:** Treatment-related organ weight differences when compared to controls (%)

Necropsy	Day 11 (one-dose groups)				Day 85 (three-dose groups)			
Sex	Male		Female		Male		Female	
Group	2	3	2	3	5	6	5	6
Treatment	MV1-F4	Rouvax	MV1-F4	Rouvax	MV1-F4	Rouvax	MV1-F4	Rouvax
Body weight (%)	+10	−1	−5	−11	+3	−8	−9	+8
Spleen								
Absolute (%)	+36	+53	+7	−6	+18	+7	+2	+72
Relative (%)	+23	+45	+14	+6	+13	+16	+12	+60*
Cervical LN								
Absolute (%)	+52	+16	−21	−40	+81	+146	−2	+7
Relative (%)	+37	+26	−15	−33	+69	+167	+6	−3
Iliac LN								
Absolute (%)	−4	+11	−9	+4	+33	+36	−40	+34
Relative (%)	−9	+20	0	+17	+30	+47	−34	+24
Thymus								
Absolute (%)	−20	−26	+19	−7	−8	−42	+4	−24
Relative (%)	−27	−23	+29	+7	−12	−39	+15	−28

Results are differences (expressed as percentages) of the mean weight of three animals per vaccine group, as compared to those of control groups. Relative organ weight = organ weight/body weight

*LN* lymph nodes

*Statistically different (*p* < 0.05) from control groups (with significance based on values, not on percentages)

At day 85, mean absolute and relative spleen weights of both vaccine groups were higher than those of controls for both sexes, which reached statistical significance (*p* < 0.05) in the Rouvax-treated females. Mean absolute and relative weights of cervical and iliac lymph nodes tended to be higher in both groups of vaccinated males as compared to the control groups, while a high variability in values were noted for the female groups. Mean thymus weights tended to be lower in both groups of vaccinated males and in Rouvax-treated females, as compared to control groups.

None of these differences between groups or sexes, observed on either day of necropsy, were supported by macroscopic observations.

For the majority of immunized animals that were sacrificed at days 11 or 85, the increase in the absolute and/or relative spleen weights could not be directly correlated to the detection of viral RNA in these spleens in the biodistribution analyses. An increased spleen weight was noted in some animals for which no viral RNA was detected in the spleen, and conversely, no increased spleen weight was observed in some animals for which viral RNA was detected in the spleen.

Microscopically, enlargements of the white pulp in the spleen were occasionally noted in the animals sacrificed at day 11, in one of the three males and one of the three females treated with MV1-F4, and in one of the three control females. This enlargement was primarily due to generation of germinal centers, and was also noted in the animals sacrificed on day 85 (in one of the three MV1-F4-treated males and two of the three Rouvax-treated males). At both time points, the increased sizes of the white pulp and/or germinal centers were not necessarily linked to the detection of viral RNA in the spleens. In addition, no enlargement was observed in some animals for which viral RNA was detected in the spleen. Furthermore, minimally increased germinal center sizes and/or numbers were occasionally noted in mandibular lymph nodes in two of the three MV1-F4-treated males sacrificed on day 11. Although this was also seen in one of three control females sacrificed on day 85, a relationship to treatment could not be excluded. This effect was rarely seen in iliac, cervical or popliteal lymph nodes. The higher absolute and relative weights of cervical and iliac lymph nodes recorded in males of both vaccine groups on day 85 did not correlate with any consistent microscopic changes.

No relevant microscopic changes were observed at the injection sites on both days of necropsy. All other microscopic findings occurred at equal frequency across groups and/or were within the normal laboratory ranges for this species, and were therefore considered as toxicologically irrelevant. Moreover, no effects suggestive of MV infection (follicular necrosis within the hair follicles, proliferative and necrotizing bronchointerstitial pneumonia, thymus atrophy or Warthin–Finkeldey cells within the lymph nodes or spleen) were found. In addition, there was no evidence of subacute sclerosing panencephalitis (a rare syndrome in humans occurring after infection with wild-type MV stains, characterized by progressive gliosis, demyelination and neuronal loss).

## Discussion

In order to support the clinical development of the MV1-F4 vaccine, we have compared the toxicology, biodistribution and shedding profiles of the HIV-1 candidate vaccine MV1-F4 to those of the parental Schwarz vaccine strain, using a cynomolgus macaque model. In addition, the results may serve to increase our knowledge of the toxicity or in vivo tropism of MV vectors or vaccine strains, which has been the focus of few preclinical studies to date (e.g., de Vries et al. 2010; Lemon et al. 2011; Myers et al. 2007; Peng et al. 2003).

Our data show that no shedding of infectious virus was observed for either of the two vaccines. Furthermore, no toxic effect in relation to the MV vaccination was found with these vaccines. One or three IM injections of a full human dose of MV1-F4 or of the reference vaccine were well tolerated and induced no clinical symptoms of local or systemic reactogenicity. For both vaccines, virus replication was predominantly observed in secondary lymphoid organs (including spleens, Peyer’s patches and all major lymph nodes) and to a lesser extent in epithelium-rich tissues (including intestine, larynx, trachea and urinary bladder) and liver, which were thus designated as potential target organs for intrinsic toxicity of the vaccines. However, no gross or histopathological effects indicative of toxicity were observed in these target organs, or in any other organs. Mean spleen weights were increased after three doses of either vaccine, which corresponded in some animals with enlargements of the white pulp, due to generation or growth of germinal centers. This effect likely resulted from immune activation of the secondary lymphoid organs by the vaccines, which has also been observed in other preclinical studies (Sheets et al. 2006; Speijers et al. 1988). It is less likely that local virus replication in these organs has caused the increased spleen weights, as no clear relationship between increased spleen weights, enlargements of the white pulp and the presence of viral RNA in the spleens could be established. All other observations were considered to be unrelated to treatment, as they occurred incidentally, without correlation between genders, or at similar frequencies in control and immunized animals.

The occasional reductions in food consumption had no impact on body weights and were not considered to be indicative of systemic toxicity. For DNA plasmid vaccines, it has been observed that food consumption data and body weights did not correlate with toxicity or other findings (Sheets et al. 2006). A similar conclusion could potentially be drawn for recombinant live-attenuated vaccines such as MV1-F4.

The only clinical pathological finding for MV1-F4 treatment groups was a slight but statistically significant decrease of the aPTT (but not of the PT) for males on day 56. However, shortening of the aPTT has generally little clinical relevance and may be associated with suboptimal sample collection or processing (Adcock et al. 1998; Awad et al. 2006). Therefore this is not considered indicative of coagulation abnormalities of toxicological relevance. We also observed that on day 85, the mean CK activity for Rouvax-treated males was significantly higher than for control males. Elevated CK values are used as a marker of injury, heart attack, severe muscle breakdown, muscular dystrophy or acute renal failure (Lott and Abbott 1986). None of these conditions were observed for the two males involved, and urinalysis showed no treatment-related effect for either vaccine. In addition, no elevated CK activity was found in these animals on previous time points. Possibly these elevations reflect the effects of IM-delivered sedation for ophthalmology on day 84. This effect is commonly seen in preclinical studies (Stewart et al. 2008) and is therefore not considered to be an adverse reaction to treatment.

Cell tropism for vaccine and wild-type MV strains is largely determined by virus entry (Tatsuo et al. 2000a). Wild-type MV, which use predominantly SLAM/CD150 expressed on activated immune cells (Tatsuo et al. 2000b), is known to mainly replicate in lymphoid organs and epithelial tissues such as the skin, respiratory tract, intestine and urinary bladder (Griffin 2007; Takeda 2008). Recent studies suggest that after aerosol infection, wild-type MV initially targets macrophages and dendritic cells in alveolar tissues, which is followed by replication in regional lymph nodes and systemic spreading to lymphoid organs (Lemon et al. 2011). How MV infects CD150-negative epithelial cells remains largely unclear, although putative epithelial receptors have recently been described (Watanabe et al. 2010). As MV vaccine strains use the ubiquitous CD46 receptor in addition to the receptors used by wild-type MV (Dorig et al. 1993), the vaccine strains were expected to exhibit a wider tropism than that documented previously for wild-type MV (Griffin 2007; Lemon et al. 2011; Takeda 2008). However, in macaques infected intratracheally or by aerosol with wild-type or attenuated (Edmonston tag) recombinant MV, only the pathogenic MV caused significant viremia and widespread distribution. The attenuated MV had a restricted systemic spread and was rarely detected in lymphoid tissues (de Vries et al. 2010), in contrast to observations in the current study. Our data seem to be more aligned with studies in mice immunized intraperitoneally or intravenously with an attenuated oncolytic (Edmonston) recombinant strain, in which infected cells were mainly detected in secondary lymphoid organs, liver and lungs (Myers et al. 2007; Peng et al. 2003). These disparities between studies may be explained by differences in administration routes, MV strains or methods of MV detection.

The RT-qPCR results for tissues and biological fluids confirm a peak of replication around 10 days after the first dose, supporting other studies in monkeys with wild-type or attenuated MV strains (Auwaerter et al. 1999; Pan et al. 2005; Permar et al. 2003). This resembles the clinical situation, as vaccine-induced peaks are known to occur a few days before those induced by natural infection taking place between 11 and 14 days after exposure (Strebel et al. 2004). We observed that viral clearance for both vaccines was not fully completed at 28 days after the third dose, since viral RNA was still detectable in lymphoid and epithelium-rich tissues. This is consistent with an earlier report showing that MV RNA remained detectable in monkeys for 4–5 months (in this case, in PBMCs), even though clearance of viremia occurred within 14 or 29 days (Pan et al. 2005).

An additional study objective was to assess the potential of virus shedding of the MV1-F4 vaccine (in parallel to that of the reference vaccine). In natural infection, MV can be isolated from urine up to 10 days after rash onset (Gresser and Katz 1960), and viral RNA can be detected in PBMC and nasopharingeal specimens up to 100 days after rash onset (Riddell et al. 2007). The few known studies of shedding by attenuated MV vectors report detection of (Edmonston) viral RNA in buccal swabs from monkeys (after intravenous administration; Myers et al. 2007), but no detection in human urine or saliva (after intraperitoneal administration; Galanis et al. 2010). Consistent with reports of detection of viral RNA from attenuated MV vaccine strains in human urine up to 14 days after vaccination (Rota et al. 1995), we detected MV1-F4-derived RNA in urine at day 11. Infectious viral particles from MV vaccines have rarely been found in human throat or nasopharyngeal secretions, and there is only one reported case of isolation of (Schwarz) MV vaccine virus from throat swabs (Morfin et al. 2002). While we detected viral RNA from both vaccines at the peak of viremia in a few samples such as throat and nasopharingal swabs, none of these contained infectious virus at a LOD of 10 CCID_50_/well (with 95 % probability). Therefore, transmission of MV1-F4 virus between persons is considered unlikely to occur, and has also never been documented previously for any of the current measles vaccines (WHO 2009).

The immunogenicity of the MV1-F4 candidate vaccine was not investigated in detail in the current study, as our study was primarely designed to assess the toxicology, biodistribution and shedding profiles of the candidate vaccine. However, immune responses directed against the F4 transgene were characterized in a separate study using the same animal model (MV-seronegative cynomolgus macaques). In the latter study, the MV1-F4 candidate vaccine was shown to induce F4-specific CD4^+^ and CD8^+^ T cell responses, as well as antibody responses to F4 (manuscript in preparation).

In conclusion, while viral dissemination was observed in various organs, no shedding of infectious viral particles was noted, and no toxic effect in relation to the MV vaccination was found following one or three IM injections with a clinically relevant dose of MV1-F4, with the same outcomes for the (Schwarz) measles comparator vaccine. Moreover, either vaccine virus replicated predominantly in secondary lymphoid organs and, to a lesser extent, in epithelium-rich tissues. Thus, as expected, introduction of the F4 transgene did not change the toxicological profile, shedding capacity or tropism of the parental strain.
